# Enhancement of NK Cell Cytotoxic Activity and Immunoregulatory Effects of a Natural Product Supplement Across a Wide Age Span: A 30-Day In Vivo Human Study

**DOI:** 10.3390/ijms26072897

**Published:** 2025-03-22

**Authors:** Sergei Boichuk, Aigul Galembikova, David Vollmer

**Affiliations:** 1 Department of Pathology, Kazan State Medical University, Kazan 420012, Russia; ailuk000@mail.ru; 2 Central Research Laboratory, Kazan State Medical University, Kazan 420012, Russia; 3 Scientific Research Division, 4Life Research, Sandy, UT 84070, USA; davidv@4life.com

**Keywords:** bioactive compounds, AgePro, NK cells, activation, cytokines, K562-based cytotoxicity, CD107a degranulation assay

## Abstract

The purpose of this study was to examine whether supplementation of ultra- and nanofiltered colostrum-based products, combined with egg yolk extract, nicotinamide mononucleotide (NMN), quercetin, alpha-ketoglutarate, white button mushroom, and celery seed extracts (the formula was patented by 4Life Research Company, USA and named as AgePro), modulate the functional activity of natural killer (NK) cells in vivo. We found that this supplement, taken orally in two capsules twice a day for 30 days, significantly enhanced the cytotoxic activity of NK cells. This was evidenced by the increased NK cell-mediated killing of carboxyfluorescein diacetate succinimidyl ester (CFSE)-labeled K562 human myeloid leukemia cells. As expected, this effect was dependent on the ratio between the effector (E) (e.g., peripheral blood mononuclear cells (PBMCs)) and target (T) (e.g., K562) cells, illustrating maximal killing of K562 cells at a 50:1 E/T ratio. Of note, increased NK-mediated killing of K562 cells after taking AgePro correlated with increased perforin release, evidenced by the CD107a degranulation assay. In concordance with these findings, taking of AgePro for 1 month increased production of several cytokines and chemokines, including IL-1β, IL-1Rα, IL-6, IL-8, IL-10, IFN-γ, TNF-α, G-CSF, PDGF-AA, PDGF-AB/BB, GRO, MCP-1, MCP-3, and MIP-1α, in PBMCs co-cultured with K562 cells. Of note, increased production of the cytokines correlated with the activation state of PBMCs, as evidenced by increased expression of the surface activation markers (e.g., the interleukin-2 receptor alpha chain—CD25). A strong correlation was found between NK-based cytotoxic activity and the production of IL-1β, IL-6, TNF-α, and MIP-1α. Importantly, no increase in the aforementioned soluble factors and activation markers was detected in PBMCs cultured alone, thereby illustrating the potent immunoregulatory activity of AgePro only in the presence of the harmful target cells. Hematological parameters also remained unchanged over the entire study period. Collectively, we show herein the significant enhancement of the cytotoxic activity of NK cells against target tumor cells after taking AgePro for 1 month. Notably, this effect was observed for all age groups, including young, adult, and elderly participants. Moreover, a significant improvement in NK cytotoxic activity was also detected for participants with low basal (e.g., before taking AgePro) numbers of NK-mediated killing. The enhancement of NK-based cytotoxicity was associated with an increased release of several cytokines and chemokines involved in regulating a broad spectrum of mechanisms outside the cell-mediated cytotoxicity and killing of target cells. Of note, spontaneous activation of PBMCs, particularly NK cells, was not detected after taking AgePro. Given that spontaneous activation of autoreactive lymphocytes is a feature associated with autoimmunity and taking into account our data illustrating the AgePro-induced activation of NK cells detected only in the presence of the potentially harmful cells, we conclude that our innovative product exhibits potent immunoregulatory activity and high safety profile.

## 1. Introduction

The role of bioactive compounds in modulating the activity of the immune system is an emerging field of clinical significance, as it highlights the close interplay between nutrients and the efficacy of innate and adaptive immune responses, which is pivotal in preventing a broad spectrum of human diseases. Indeed, apart from interleukins, which belong to the best-characterized group of compounds exhibiting immunostimulatory capacities, nutrients and constituents extracted from plants also display the ability to stimulate and/or modulate the activity of diverse immune cells, including NK cells, T- and B-lymphocytes, dendritic cells, macrophages, neutrophils, etc. In particular, the activity of NK cells might be effectively regulated by vitamins A, B, C, D, and E [1,2,3,4,5,6,7], polysaccharides, lectins, and several phytochemicals (e.g., genistein, curcumin, resveratrol, quercetin, etc.) [8,9,10,11], which results in increased NK-mediated killing of target cells, higher levels of their degranulation, and/or enhanced IFN-γ and TNF-α production [reviewed in more detail in [12]. In particular, apart from the well-known antioxidative, anti-inflammatory, and neuroprotective activities, the flavonol compound quercetin was shown recently to increase the proportion and maturation of NK cells without affecting T or B cells, thereby improving cognitive performance in mice. In contrast, the depletion of NK cells significantly reduced their cognitive abilities [11]. Moreover, quercetin was proposed to mitigate the immunosenescence hallmarks in human PBMCs in vitro by restoring the proliferative capacity of aged cells and increasing their iNOS activity assessed by nitric oxide release [13], thereby suggesting senolytic activity of this bioactive supplement.

Similarly, NMN, a key nicotinamide adenine dinucleotide (NAD+) intermediate, was shown to mitigate age-associated physiological decline in mice on multiple levels, including age-associated body weight gain, enhanced energy metabolism, improved insulin sensitivity, and plasma lipid profile [14]. The results of placebo-controlled, randomized, double-blind trials clearly illustrated that oral supplementation of NMN (250 mg/day) for 12 weeks significantly increased NAD+ levels in whole blood without obvious adverse effects and abnormalities in physiological and laboratory tests [15]. Additionally, NMN has also been shown to augment NK cell cytotoxic activity in preclinical and clinical models. For example, oral administration of NMN increased NK-based cytotoxicity in young and elderly mice without affecting the number of NK cells. Of note, NMN did not enhance NK-mediated cytotoxic activity in IFN-γ deficient mice, suggesting that NMN administration augments NK cell cytotoxic activity in an IFN-γ-dependent manner [16]. Of note, NK cells cultured in the presence of NAD exhibited potent metabolic changes associated with elevated glucose flux and protection against oxidative stress. As expected, NAD-treated NK cells displayed enhanced cytotoxicity and inflammatory cytokine production. Moreover, NK cells cultured with NAD and interleukin-15 (IL-15) exhibited the increased expression of L-selectin (CD62L), a well-known lymphocyte adhesion molecule playing a crucial role in lymph node homing. Strikingly, in a phase 1 trial of NAD-expanded allogeneic NK cells, rituximab (the monoclonal antibodies targeting CD19 molecule), and IL-2, 11 of 19 patients with relapsed or refractory non-Hodgkin lymphoma (NHL) and multiple myeloma (MM) (NCT03019666) demonstrated complete response, suggesting that supplementation with NAD during ex vivo NK cell culture should be further investigated in detail for NHL therapy [17].

Besides the essential immunomodulatory role of the aforementioned bioactive compounds, bovine colostrum comprising a broad spectrum of bioactive molecules, including immunoglobulins and antimicrobial peptides, was also shown to effectively modulate the functional activities of both (e.g., innate and adoptive) branches of immunity [18]. In particular, ultrafiltered bovine colostrum and hydrolyzed whey significantly increased NK activity in vivo, which was evidenced by an increased production of cytokines (e.g., IL-2, IFN-γ, and TNF-α) after their oral administration to mice [19,20,21]. Our recent study also demonstrated a high immunomodulatory potency of colostrum-based natural products, whey extract, and their combinations with egg yolk extract and mushroom extracts on PBMC. Indeed, we observed an increased production of cytokines in lipopolysaccharide (LPS)-treated PBMCs cultured in the presence of colostrum-based natural products when compared with control (e.g., PBMCs cultured with LPS alone). In contrast, a decreased amount of cytokines was found in the supernatants of phytohemagglutinin (PHA)-activated PBMCs cultured in the presence of colostrum-based natural products, thereby illustrating their potent immunomodulatory activities and ability to prevent excessive activation of immune cells [22].

The present study aimed to examine the immunomodulatory activities of the ultra- and nanofiltered colostrum-based natural products combined with egg yolk extract, NMN, quercetin, alpha-ketoglutarate, white button mushroom, and celery seed extracts. The 4Life Research Company, USA, patented this formula and named AgePro. This product was given orally to healthy participants to assess its immunomodulatory activity in vivo. We showed here for the first time that taking this supplement in two capsules twice a day for 30 days significantly improved the cytotoxic activity of NK cells. This was evidenced by an increase in PBMC-mediated killing of carboxyfluorescein diacetate succinimidyl ester-labeled (CFSE-labeled) K562 human myeloid leukemia cells. As expected, this effect was ratio-dependent and illustrated the maximal killing of K562 target (T) cells by PBMC effector (E) cells used at a 50:1 E/T ratio. Of note is that the NK-mediated killing of K562 cells correlated with NK-based degranulation, as evidenced by the CD107a degranulation assay. Lastly, AgePro significantly increased the production of several cytokines (e.g., IL-1β, IL-1Rα, IL-6, IL-8, IL-10, TNF-α, G-CSF, etc.) and chemokines (e.g., GRO, MCP-1, MCP-3, MIP-1α, etc.) by PBMCs co-cultured in the presence of K562 cells. Importantly, no evidence of spontaneous activation of PBMCs was found in the absence of K562 target cells, thereby highlighting the immunomodulatory potency of AgePro.

Collectively, we showed a significant increase in NK-based cytotoxic activity after taking AgePro as an oral supplement for 1 month. This effect was observed for all age groups, including young and adult participants (age < 35 and >50, respectively). Moreover, a substantial improvement in NK cytotoxic activity was detected for the participants with very low basal (e.g., before AgePro) PBMC-mediated cytotoxicity levels, illustrating this bioactive supplement’s potent immunomodulatory activity.

## 2. Results

### 2.1. AgePro Uptake Increases the Cytotoxic Activity of NK Cells and Enhances K562 Killing In Vitro in a Ratio-Dependent Manner

**Figure 1:**
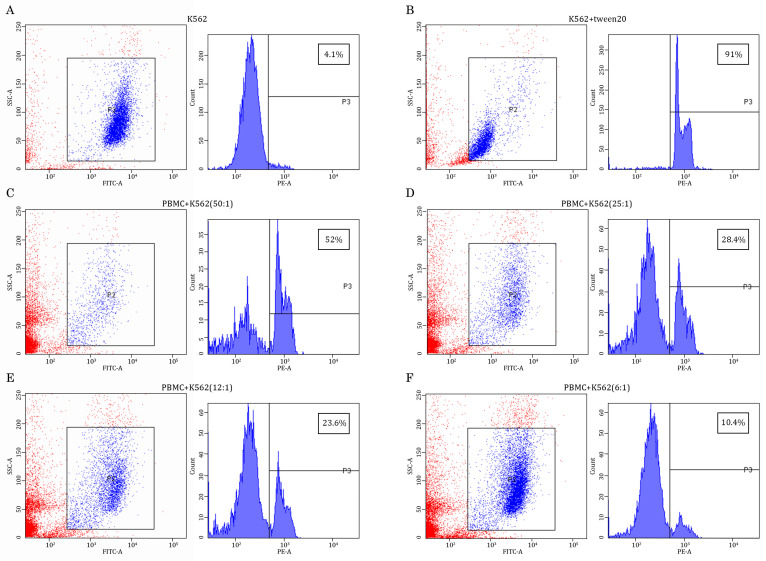
Assessment of K562 lysis in PBMC/K562 cultures. K562 target cells were selected from PMBC/K562 cultures based on the CFSE-induced intensity, which was examined on the FL1 channel (FITC-A). These cells were gated in the R2 region, as shown in the blue in the multi-color plots. The PBMCs exhibiting low intensity of CFSE stain are shown in red color. Next, the histograms were generated for the R2 region to examine the intensity of propidium iodide staining, which was assayed on the FL2 channel (PE-A) and used as a marker of cell lysis (shown in percentages on the upper right corner of each histogram). K562 cells cultured alone were used as a negative control (**A**), whereas K562 cells exposed to Tween-20 served as a positive control of cell lysis, which was detected by FACs staining due to increased uptake of propidium iodide (**B**). Effector (E) PBMCs were cultured with K562 target (T) cells were co-cultured at different E:T ratios (e.g., 50:1; 25:1; 12:1; 6:1) (**C**–**F**) to illustrate dose-dependent cytotoxicity of PBMCs against K562 tumor cells.

Given that the primary goal of the study was to examine whether the AgePro supplement taken orally in two capsules twice a day for 30 days has an impact on the cytotoxic activity of NK cells, we performed K562-based cytotoxicity assays according to the standard protocol, as shown elsewhere [23]. For this purpose, we used purified PBMCs and co-cultured them with K562 target cells pre-labeled with carboxyfluorescein diacetate succinimidyl ester (CFSE), which is a stable, protein-binding fluorescent dye that allows for discrimination of target cells from effector NK cells [24]. K562 cells cultured alone were used as a negative control (Figure 1A), whereas K562 cells exposed to Tween-20 served as a positive control of cell lysis, which was detected by FACs staining due to increased uptake of propidium iodide (Figure 1B). Different ratios between effector (E) and target (T) cells were used. As expected, maximal killing of K562 cells was observed for the highest (e.g., 50:1) E:T ratio (Figure 1C), whereas lower E:T ratios (e.g., 25:1; 12:1; 6:1) exhibited less amount of the lyzed K562 target cells (Figure 1D, Figure 1E and Figure 1F—respectively).

**Figure 2:**
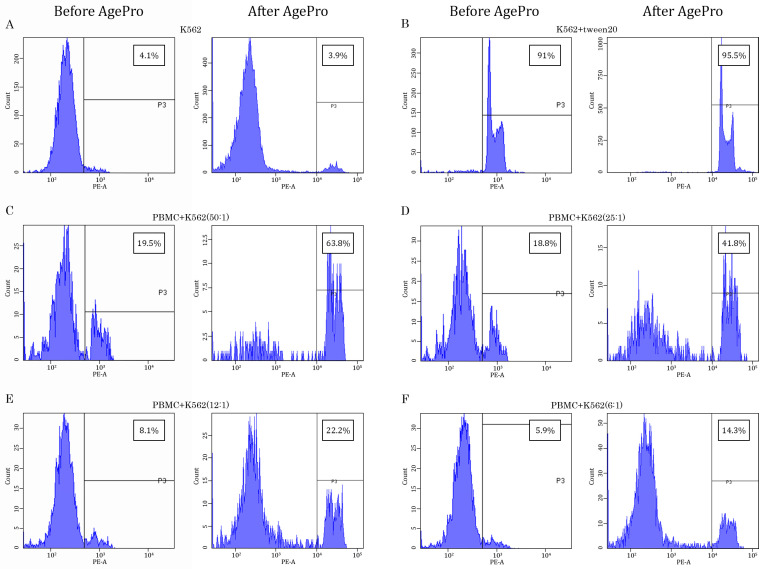
AgePro increases the PBMC-mediated lysis of K562 target tumor cells. Representative histograms illustrating the intensity of propidium iodide (PI)-based fluorescence of K562 target cells cultured alone (negative control) (**A**), in the presence of Tween-20 (**B**) (positive control), or the presence of PBMCs used at the different ratios with K562 cells (**C**–**F**). The histogram contains the samples before and after taking AgePro for 30 days. The increased PI-based fluorescence was used as a marker of lysis of K562 cells calculated in %, as shown in the quadrants on the upper right corner of each histogram.

**Figure 3:**
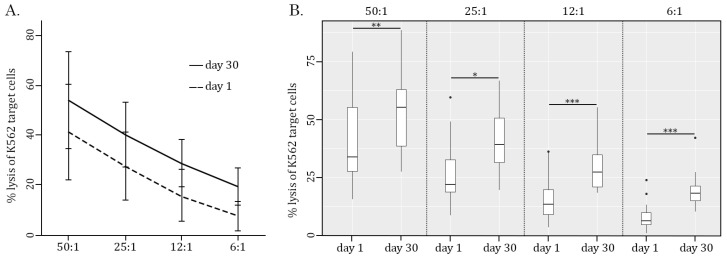
Ratio-dependent cytotoxicity of PBMC effector (E) cells against K562 target (T) cells. (**A**) PBMC-mediated cytotoxicity against CFSE-labeled K562 tumor cells was assessed at various E:T ratios (50:1, 25:1, 12:1, and 6:1) and determined by FACs staining. (**B**) Boxplots represent the cytotoxicity of PBMCs against K562 cancer cells cultured at various E:T ratios (50:1, 25:1, 12:1, and 6:1). Number of participants (n = 17). Statistical significance between 2 samples (day 1/day 30) was determined by paired Student’s *t*-test (in the case of normally distributed data) or Wilcoxon signed-rank test (in the case of not normally distributed data). All data are shown as mean ± SD (**A**) and median (quant 25—quant 75) (**B**). * *p* < 0.05, ** *p* < 0.01, *** *p* < 0.0001. To further corroborate these findings and illustrate whether the efficacy of AgePro does not depend on the age of the participants, they were divided into two groups based on the increased value of K562 cell killing after taking AgePro (e.g., enhancement of K562 cell lysis for less or more than 20%, respectively). Using Fisher’s exact two-sided test, we found similar efficacy in NK-based killing of K562 for both groups. Again, this was shown for all E:T ratios utilized in this study (*p* > 0.05). Strikingly, a substantial improvement in NK cytotoxic activity was also detected for the participants with low basal (e.g., before AgePro) numbers of PBMC-mediated killing, thereby illustrating the potent immunomodulatory activity of this supplement.

Strikingly, the AgePro supplement taken orally in two capsules twice a day for 30 days significantly increased the cytotoxic activity of NK cells. The representative histograms in Figure 2 illustrate the significant increase in K562 killing after taking AgePro compared to the baseline (e.g., before taking AgePro). As shown in Figure 2 and Figure 3, enhanced killing of K562 cells was detected for all E:T ratios. Importantly, such an effect was found for all age groups (e.g., <35 and >50 years old participants), as shown in Table 1.

### 2.2. AgePro Increases CD107a Expression in NK Cells

Given that CD107a (LAMP-1) is a molecule expressed on activated NK and CD8+ T cells, and considering that functional CD107a is required for efficient perforin delivery to lytic granules and NK-mediated cytotoxicity [25], we further examined whether the AgePro supplement, taken orally in two capsules twice a day for 30 days, increases the expression of this molecule on NK cells cultured in the presence of K562 cells. PBMCs cultured alone were used as a negative control, whereas PBMCs cultured with IL-2 (20 ng/mL) were used as a positive control.

**Figure 4:**
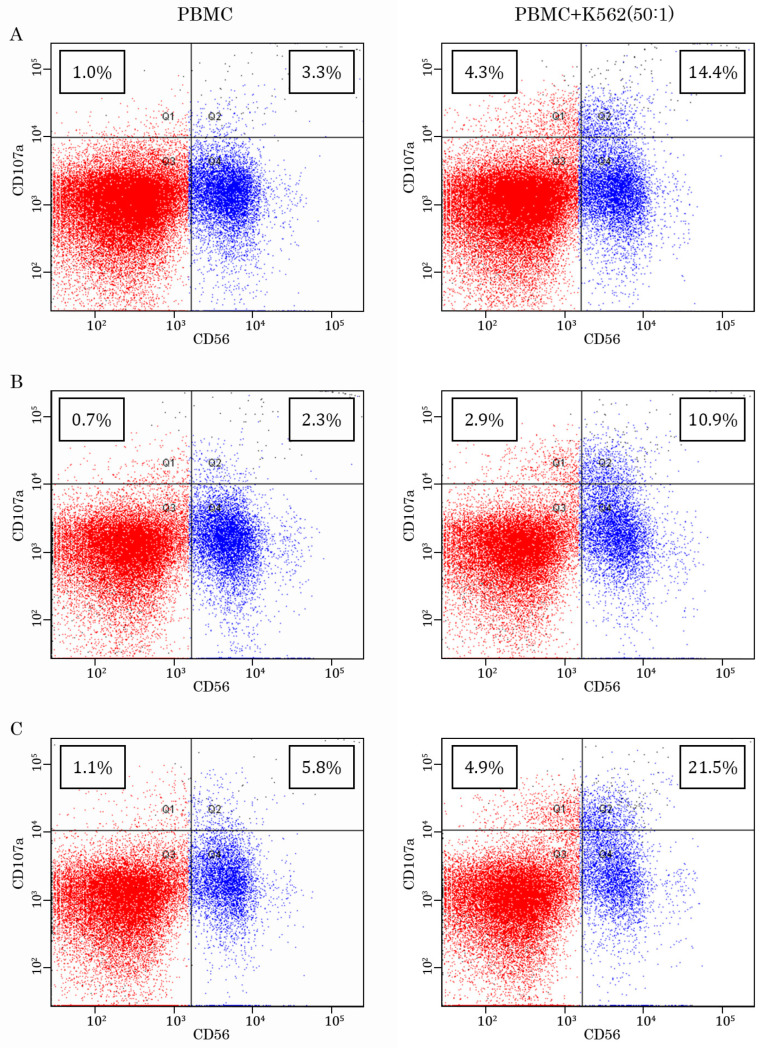
Representative dot-plots illustrating the expression of CD107a molecule on the CD56-positive cells. Data is shown for 3 participants ((**A**–**C**), respectively). (**Left panel**)—PBMCs cultured alone, (**right panel**)—PBMCs cultured in the presence of K562 target tumor cells.

We initially examined whether the co-culture of PBMCs with K562 target cells increases CD107a expression in NK cells. As shown in Figure 4A–C—left panel, in PBMCs cultured alone, CD56-positive cells exhibited a low expression of the CD107a marker, whereas the presence of K562 target cells significantly increased the number of double-positive cells (e.g., CD56/CD107 cells) (Figure 4A–C—right panel), thereby illustrating an overall increase in degranulation in PBMCs cultured with K562 target cells. Of note, we also detected a minor increase in the number of CD56−/CD107a+ cells in PBMC/K562 co-cultures when compared with PBMCs alone, thereby suggesting the activation and degranulation beyond NK cells. This was in concordance with the literature data illustrating the expression of CD107a degranulation marker in diverse populations of white blood cells, including CD8+ T cells [26].

Table 2 illustrates the increased numbers of CD107a-positive cells among PBMCs cultured in the presence of IL-2 or K562 cells or in combination compared to baseline numbers.

**Figure 5:**
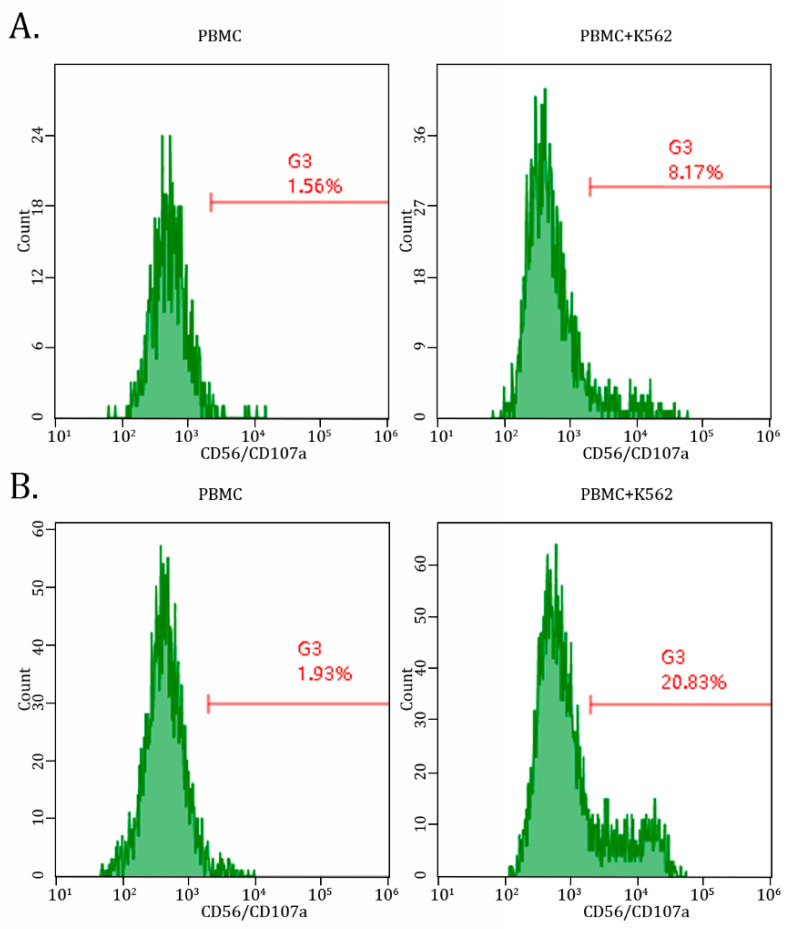
AgePro increases NK-based degranulation in PBMCs cultured with K562 target cells. Representative histograms illustrate the expression of CD107a molecules in PMBCs cultured with K562 target cells (**right**) when compared with PBMCs alone (**left**). Data shown in (**B**) illustrates increased CD107-positive cells after taking AgePro compared to the AgePro-naive sample (**A**).

Next, we found a significant (~5-fold) increase in double-positive (e.g., CD56/CD107a) cells after taking AgePro. Importantly, this was observed only in PBMCs cultured in the presence of K562 target cells, whereas no increase in degranulating NK cells was detected in PBMCs cultured alone (Figure 5A,B).

Overall, this data suggests that increased cytotoxic activity of NK cells after taking AgePro is related to enhancing their degranulation, as evidenced by increased numbers of CD107a-positive cells.

### 2.3. AgePro Stimulates the Production of Cytokines/Chemokines by PBMCs Cultured with K562 Target Cells

**Figure 6:**
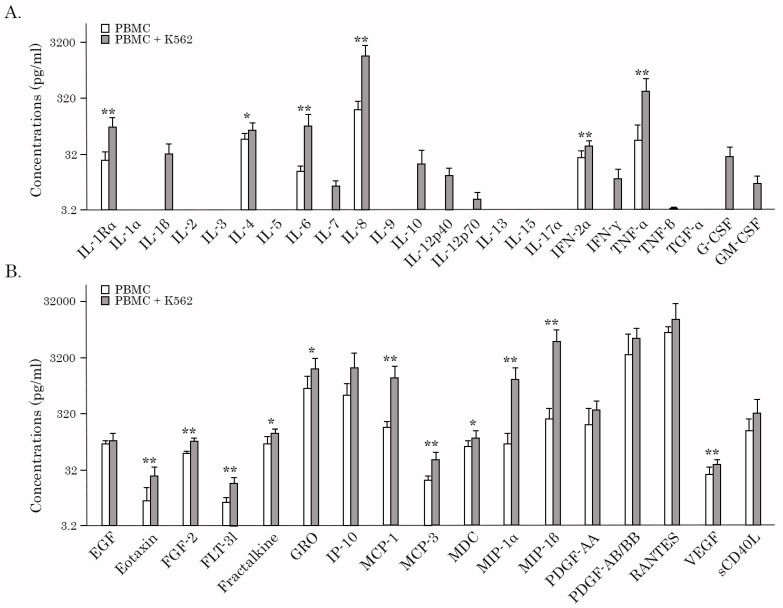
The concentration of cytokines and chemokines (pg/mL) in the supernatants of PBMCs cultured alone (gray columns) and in the presence of K562 target cells (white columns) (ratio 50:1) before taking AgePro. (**A**) IL-1Rα, -1α, -1β, -2, -3, -4, -5, -6, -7, -8, -9, -10, -12p40, -12p70, -13, -15, and -17α, INF-2α, INF-γ, TNF-α, TNF-β, TGF-α, G-CSF, and GM-CSF; (**B**) EGF, eotaxin, FGF-2, FLT-3l, fractalkine, GRO, IP-10, MCP-1, MCP-3, MDC, MIP-1α, MIP-1β, PDGF-AA, PDGF-AA/AB, RANTES, VEGF, and sCD40L. * *p* < 0.05, ** *p* < 0.01.

Next, we examined whether the AgePro supplement can modulate the production of cytokine/chemokines by PBMCs cultured with K562 target cells in 200 μL culture media at 50:1 ratio for 4 h at 37 °C. PBMCs cultured alone were used as a negative control. The concentrations of 41 chemokines (MCP-1, MCP-3, MIP-1α, RANTES, GRO, VEGF, FGF, EGF, etc.) and cytokines (IL-1β, IL-2, IL-3, IL-4, IL-5, IL-6, IL-7, IL-8, IL-9, IL-10, IL-12p70, IL-15, IL-17, IFN-γ, TGF-α, TNF-α, G-CSF, GM-CSF, etc.) in culture supernatants were measured with an MILLIPLEX MAP human cytokine/chemokines panel (Merck, Kenilworth, NJ, USA). Initially, we examined whether PBMCs released the aforementioned soluble factors upon four hours of incubation with K562 target cells. As anticipated, stimulation of NK cells by K562 target cells increased the production of IL-1Rα, IL-4, -6, and -8, IFN-2α, and TNF-α (Figure 6A, Supplementary Table S1). Additionally, IL-1β, IL-7, -7, -10, and -12, IFN-γ, and both colony-stimulating factors (e.g., G-CSF and GM-CSF) were found in PBMC/K562 supernatants only (Figure 6A, Supplementary Table S1). Of note, the levels of several cytokines, including IL-1Rα, IL-6, and IFN-2α in PBMCs cultured alone, were very low, thereby illustrating K562-induced stimulation of PBMCs, including NK cells. Additionally, K562-induced stimulation of NK cells enhanced the secretion of several chemokines, including eotaxin, FGF-2, GRO, MCP-1, MCP-3, VEGF, and MIP-1α (Figure 6B, Supplementary Table S1). Again, much lower levels of the chemokines mentioned above were detected in the supernatants of PBMCs cultured in the absence of K562 target cells, suggesting a degree of constitutive secretion. Lastly, IL-2, -3, -5, -9, -13, -15, and -17α and TGF-α were not detected in PBMCs and PBMC/K562 cultures (Figure 6A,B).

Given that the production and secretion of cytokines and chemokines is a signature of cell activation, we also examined the expression of activation markers, including the CD25 molecule, a well-known receptor for the alpha chain of IL-2, and HLA-DR late activation marker, on PBMCs. As shown in Supplementary Figure S1 and Supplementary Table S2, we observed a ~60% increase in CD25-positive cells in PBMCs cultured in the presence of K562 target cells compared with PBMCs alone, thereby illustrating K562-induced activation of mononuclear cells. In contrast, no increase in the number of HLA-DR-positive cells in PBMC/K562 cultures was observed, probably due to their co-cultures short period (12 h). As expected, PHA-stimulated PBMCs that served as a positive control for these experimental settings exhibited a substantial increase in CD25-positive cells (Supplementary Figure S1A).

**Figure 7:**
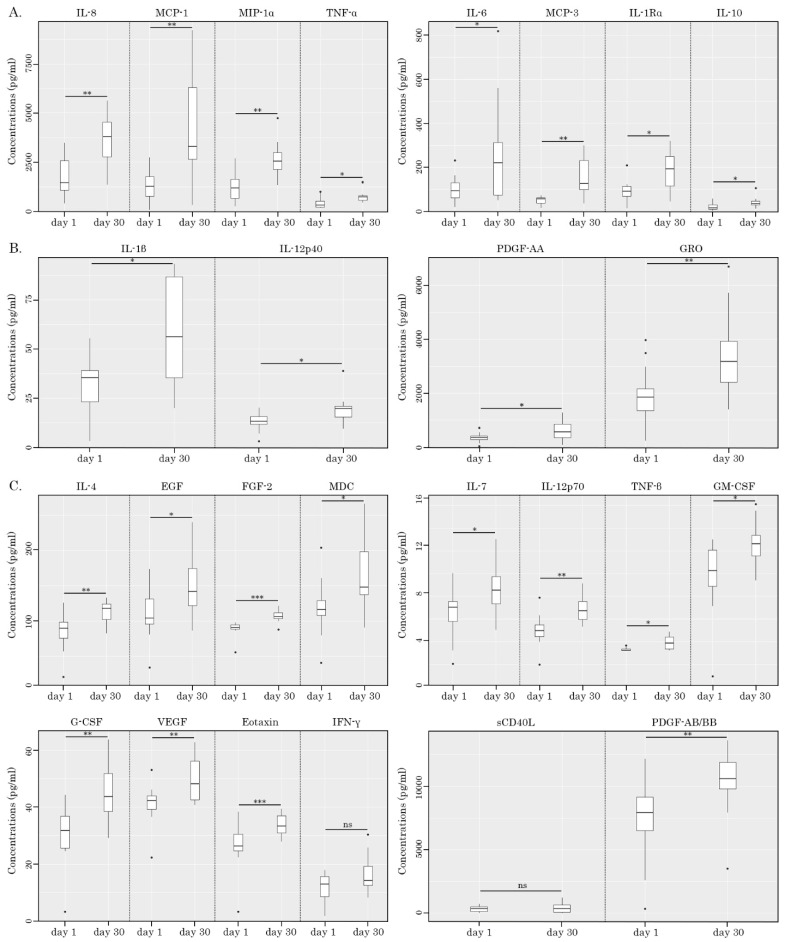
Changes in cytokine/chemokines levels in PBMC/K562 supernatants before (Day 1) and after taking AgePro (Day 30). Boxplots illustrate concentrations of cytokines/chemokines, as shown in pg/mL. (**A**) IL-8, MCP-1, MIP-1α, TNF-α, IL-6, MCP-3, IL-1Rα, and IL-10 are exhibiting >100% increase after taking AgePro; (**B**) IL-1β, IL-12p40, PDGF-AA, and GRO are exhibiting >50% increase after taking AgePro; (**C**) IL-4, EGF, FGF-2, MDC, IL-7, IL-12p70, TNF-β, GM-CSF, G-CSF, VEGF, eotaxin, INF-γ, sCD40L, and PDGF-AA/AB are exhibiting <50% increase after taking AgePro. Statistical significance between 2 samples (day 1/day 30) was determined by paired Student’s *t*-test (in the case of normally distributed data) or Wilcoxon signed-rank (in the case of not normally distributed data). All data are shown as median (quant 25–quant 75). * *p* < 0.05; ** *p* < 0.01; *** *p* < 0.001; ns, nonsignificant.

Next, we compared the levels of the aforementioned soluble factors in PBMC/K562 supernatants before and after taking AgePro. Strikingly, we observed elevated levels of 26 chemokines/cytokines in PBMC/K562 supernatants compared to the AgePro-naive samples. This included IL-1β, IL-1Rα, IL-4, -6, -7, -8, -10, and -12, TNF-α, TNF-β, G-CSF, GM-CSF, EGF, FGF-2, PDGF-AA, PDGF-AA/AB, VEGF, GRO, MCP-1, MCP-3, MIP-1α, eotaxin, and MDC. The most prominent increase (>100%) was found for IL-1Rα, IL-6, -8, and -10, TNF-α, MCP-1, -3, and MIP-1α (Table 3). This is also shown in Figure 7A. A moderate increase (>50%) after taking AgePro was observed for IL-1β, IL-12p40, GRO, and PDGF-AA (Figure 7B, Table 3). A minor increase in ranking between 20 and 50% was found for IL-4, -7, and -12p70, TNF-β, G-CSF, GM-CSF, EGF, PDGF-AA/AB, VEGF, eotaxin, and MDC (Figure 7C, Table 3). Even though the role of several cytokines, including TNF-α and IFN-γ in the NK-mediated killing of target cells is well-documented by multiple studies, we did not find a significant increase in IFN-γ in PBMC/K562 supernatants after taking AgePro (Figure 7C, Table 3). No increases in sCD40L, IP-10, MIP-1β, RANTES, FLT-3l, fractalkine, INF-α2, and -γ were also observed after taking AgePro. Lastly, IL-1α, -2, -3, -5, -9, -13, -15, and -17α and TGF-α were not detected in PBMC/K562 supernatants.

The increased levels of several chemokines in PBMC/K562 supernatants after taking AgePro (e.g., GRO, MCP-1, MCP-3, IL-8, MIP-1α, PDGF-AA, and PDGF-AA/AB (Figure 7 and Table 3)), illustrates AgePro’s ability in regulating the other processes besides NK-based cytotoxic activity, including the priming, homing, and recruitment of diverse immune cells. Despite the lack of evidence illustrating NK’s ability to produce some of these chemokines directly, the increased amounts of the aforementioned chemokines in PBMC/K562 supernatants might also be due to the NK-based activation of the other subsets of immune cells in bulk PBMC cultures. Further studies are needed to elucidate these possibilities.

### 2.4. NK-Based Cytotoxicity Correlates with Increased Production of TNF-α, IL-6, and IL-1β

**Figure 8:**
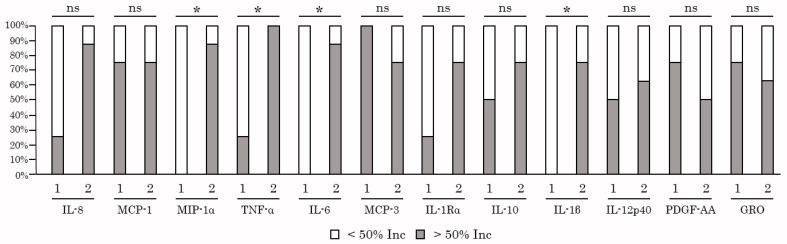
The significance of the relationship between cytokine/chemokines production (<50% and >50% Inc after taking AgePro) and NK-based cytotoxic activity was assessed by a two-tailed Fisher’s exact test. Comparison of two independent groups: group 1 with RR < 20% and group 2 with RR > 20%. *—the significance between aforementioned parameters was found (*p* < 0.05); ns—relationship was not significant.

Next, we examined whether increased levels of cytokines/chemokines in PBMC/K562 cultures detected after taking AgePro correlated with the cytotoxic activity of NK cells. For this purpose, the cytokines and chemokines were previously divided into three groups based on the increased values after taking AgePro (e.g., high > 100%, moderate between 50 and 100% increase, low—<50%), as shown in Figure 7 and Table 3. The cytokines that belonged to groups 1 and 2 were chosen for the correlation analysis. For this analysis, all the participants were also divided into two groups based on the intensity of cytotoxic response rate (RR) measured as % of the increased killing of K562 cells after taking AgePro—e.g., group 1 with RR < 20% and group 2 with RR > 20%, as shown in Figure 8. Based on this segregation approach, we found that the levels of several cytokines in supernatants of PMBC/K562 cultures correlated with NK-mediated cytotoxic activity. This belonged to IL-1β, IL-6, and TNF-α (Figure 8). Similarly, increased expression of chemokine MIP-1α after taking AgePro correlated with enhanced NK-based cytotoxicity against K562 target cells (Figure 8). In contrast, group 1 exhibited lower cytokine responses after K562-based stimulation of NK cells (Figure 8), thereby revealing the strong relationship (e.g., φ > 0.7) between NK-based cytotoxic activity and production/secretion of the cytokines/chemokines mentioned above (Supplementary Table S3). Additionally, by using Spearman’s rank correlation assay, we found a strong correlation pattern between NK-based killing of K562 cells and the production of IL-6, TNF-α, and MIP-1α (Table 4).

### 2.5. AgePro Does not Induce Spontaneous Activation of PBMC

Notably, after taking AgePro, no increase in cytokines/chemokines was observed in the supernatants of PBMCs cultured without K562 target cells (Supplementary Figure S2 and Supplementary Table S4).

This was in concordance with the low expression of activation markers (e.g., CD25 and HLA-DR) on PBMCs after taking AgePro, as was described above. Similarly, no increase in spontaneous degranulation of NK cells measured by CD107a degranulation assay was detected after taking AgePro (Figure 5 and Table 5).

Lastly, AgePro did not induce hematological changes in all participants enrolled in the present study. This included the number of red blood cells (RBCs), white blood cells (WBCs), hemoglobin (HGB), hematocrit (HCT), mean corpuscular volume (MCV), mean corpuscular HGB (MCH), mean corpuscular HGB concentration (MCHC), RBCl distribution width coefficient of variation (RDW-CV), RBC distribution width standard deviation (RDW-SD), platelets (PLTs), mean platelet volume (MPV), platelet distribution width by volume (PDW), platelet large cell ratio (P-LCR), platelet crit (PCT), immature granulocytes (IG), neutrophils (Neu), lymphocytes (Lym), monocytes (Mon), eosinophils (Eos), basophils (Bas), nucleated RBCs (NRBCs), and erythrocyte sedimentation rate (ESR) (Supplementary Table S5).

## 3. Discussion

NK cells belong to the family of innate lymphoid cells (ILCs), which play a key role in the early phase of innate immune responses against infections and malignancies due to the potent killing capacities against virus-infected and transformed cells that are dangerous to the host [27,28]. Besides infections and malignancies, NK cells also play a regulatory role in autoimmune [29,30] and metabolic diseases [31]. Indeed, this fact was proven for systemic lupus erythematosus, type 1 diabetes mellitus, and autoimmune liver disease [32,33,34]. Mechanistically, activated NK cells release perforins and granzymes to exert antibody-dependent and independent cytotoxicities and induce apoptosis to remove hostile cells. They also secrete several cytokines, including IFN-γ, IL-12, IL-15, and IL-18, thereby regulating both branches of innate and adaptive immunity [35,36]. Thus, maintaining NK cells’ appropriate functional and metabolic activities is crucial for immunosurveillance in a broad spectrum of human diseases by preventing the development and progression of cancer, viral infections, autoimmune and metabolic disorders, etc.

Besides interleukins, the well-known activators of the immune system, vitamins, and natural compounds also display potent immunomodulatory activities for T- and B-lymphocytes, dendritic cells, macrophages, neutrophils, and NK cells. Multiple reports, including our own, demonstrate immunomodulatory properties of colostrum-based products affecting the activity of the diverse components of the immune system [18,19,20,22]. In particular, we showed recently high immunomodulatory potency of modified ultrafiltrated colostrum on NK-cell cytotoxic activity against K562 target cells. Important, this data was obtained by using NK cells that were previously enriched immunomagnetically from PBMC. As we expected, granzyme B and INF-γ production highly correlated with NK cell cytotoxic activity [37]. Quercetin was shown recently to stimulate the activity of NK cells without affecting T or B cells [11] and also mitigated the immunosenescence hallmarks in human PBMCs in vitro by restoring the proliferative capacity of aged cells [13]. The results of a placebo-controlled study illustrated that the alpha-ketoglutarate (AKG)-based formulation, named Rejuvant^®^, induced an average 8-year reduction in biological aging based on the results of DNA methylation testing [38]. High anti-aging potency was also shown for white button mushroom extract (e.g., spermidine) in animal models. Besides spermidine effectively attenuated distinct age-associated markers (e.g., brain glucose metabolism, age-induced hair loss, etc.), this bioactive compound also decreased telomere attrition, thereby illustrating a novel anti-aging mechanism of this bioactive compound [39]. Moreover, our recent preclinical study demonstrated that composition, including quercetin, NMN, AKG, spermidine, and extracts from cow colostrum and chicken egg yolk, provided the potent antiaging effect in *C. elegans*, which is known as a standard model to assess improvements in lifespan and healthspan. Important, the combination of cow colostrum and chicken egg yolk extracts effectively potentiated the cytotoxic activity of PBMCs obtained from young (age < 35) and old (age > 55) participants, thereby illustrating that these compounds can provide their immunostimulatory activity in an age-independent manner [40].

Thus, the present study aimed to examine the immunomodulatory activities of ultra- and nano-filtered colostrum-based natural products combined with egg yolk extract, white button mushroom and celery seed extracts, quercetin, AKG, and NMN. This innovative formula, AgePro, was patented by 4Life Research Company, USA, and was used to assess its immunomodulatory activities of NK cells in vivo.

Most studies illustrating the low NK count and dysfunction of the NK activity (NKA) are underlying in the cancer research area. Indeed, the decreased number of NK cells and dysfunction of their activity are well-documented for a broad spectrum of human malignancies, including follicular lymphoma [41], multiple myeloma [42], a broad spectrum of solid tumors, including head and neck cancer, breast, and lung cancer, etc. [43]. NK cells were also shown to target circulating tumor cells, thereby preventing the disease progression and formation of metastatic lesions [44,45]. Besides the high prognostic values of NK counts in human diseases, the assessment of NKA provides a more precise and accurate approach to determining potential dysfunctions of these immune cells. The current approaches aimed to assess the functional state of NK cells are the following:

1. The cytotoxicity assays based on the direct measurement of the killing of previously labeled (e.g., with carboxyfluorescein diacetate succinimidyl ester-labeled (CFSE)) target cells (e.g., K562 human myeloid leukemia cells). Due to the reduced expression of HLA class I molecules present in K562 cells and increased expression of ligands for activatory NK receptors, these cells are considered the most suitable target cells to examine the functional activity of NK cells and NK cell-mediated cytotoxicity [46]. Indeed, the loss or aberrant expression of MHC-I molecules on tumor cells was shown as the most critical signal for NK cell recognition [47,48];
2. CD107a degranulation assay designed to count the amount of secreted cytotoxic granules, including perforin and granzyme, which trigger target cell death [49];
3. Assays aimed to examine the cytokines release activity. Based on these assays, impaired NKA was found to be a driving force for several human diseases. Notably, low or absent NKA is currently established as a significant characteristic of hemophagocytic lymphohistiocytosis (HLH) [50,51]. Impaired NKA was also shown for liver diseases, including hepatocellular carcinomas (HCCs) and cirrhosis [52], other types of human malignancies, and viral infections, as well.

Using these approaches, we showed here for the first time that our innovative product, AgePro, taken twice a day for 1 month, significantly improved NKA in healthy subjects. This was evidenced by the increased killing of K562 target cells, as shown in Figure 2 and Table 1. Notably, the enhancement of NK cell-mediated killing of K562 cells was evenly detected for all the participants independently from their age (Table 1). Of note, the increased lysis of K562 cells was more pronounced for the participants who initially (e.g., before taking AgePro) exhibited low NK-mediated killing of target cells. In concordance with this data, we also detected increased CD56/CD107a-positive cells in PBMC/K562 cultures, thereby illustrating the increased degranulation of NK cells. Important, AgePro induced a substantial increase in CD56/CD107a-positive cells in PBMC/K562 cultures, whereas no increase in the degranulating NK cells was found in PBMCs cultured alone (Figure 5B). These results are consistent with the literature data illustrating the CD107a molecule as a functional marker for identifying NK-based cytotoxic activity [49,53]. Interestingly, it was also shown that besides the tight relationship between NK degranulation and the expression of CD107a molecules on NK cells, these molecules might also protect these immune cells from self-destruction during degranulation-associated damage [54]. Thus, increased expression of CD107a molecule on the surface of NK cells after taking AgePro illustrates the ability of this bioactive supplement to maintain the proper functional activity of NK cells and their survival.

Next, we observed significant changes in PBMC’s secretome after taking AgePro. Noteworthy, this was evidenced only for PBMC/K562 cultures (Figure 7, Table 3), whereas the cytokine/chemokines profile in PBMCs cultured alone remained unchanged. This illustrates the absence of excessive activation of immune cells after taking AgePro and highlights the potent immunomodulatory activity of this supplement, activating the immune cells only in the presence of harmful cells. In particular, we detected elevated levels of 13 cytokines and chemokines in PBMC/K562 supernatants after taking AgePro. This included IL-1β, IL-1Rα, IL-6, -8, and -10, TNF-α, G-CSF, GRO, MCP-1, MCP-3, MIP-1α, PDGF-AA, and PDGF-AA/BB. Given that K562 cells lacking MHC class I molecules on their surfaces are known as the primary targets for NK cells, we postulated that several cytokines and chemokines that were increased in PBMC/K562 cultures were produced by activated NK cells. Indeed, K562 cells are a human myeloid cell line with reduced expression of HLA class I and increased expression of ligands for activatory NK receptors, making them susceptible to NK cell-mediated cytotoxicity [46]. Moreover, unlike adaptive T lymphocytes, NK-cell-mediated killing of target cells does not require prior priming and is independent of human leukocyte antigen (HLA) restriction. The activity of NK cells is generally regulated by the activatory and inhibitory receptors recognizing the germline-encoded molecules on the target cells [55,56].

Even though the most prominent cytokines involved in NK-based cytotoxicity are known as TNF-α and IFN-γ, the strong correlation pattern between NK-based cytotoxic activity and production was also found for IL-1β, IL-6, and MIP-1α (Figure 8, Table 4). Besides these factors, the activated NK cells have been reported to produce several other factors, including immunoregulatory cytokines such as IL-5, IL-8, IL-10, IL-13, FLT3LG, TGF-α, the chemokines MIP-1α, MIP-1β, XCL1/2, CCL3/4/5 and RANTES, and several growth factors, including GM-CSF, G-CSF, M-CSF, which recruit other immune cells and regulate their anti-tumor responses [57,58,59,60,61,62,63]. Thus, elevated TNF-α, IL-10, G-CSF, GM-CSF, and MIP-1α in PBMC/K562 supernatants indicate K562-induced activation of NK cells. In contrast, the sources of the remaining cytokines and chemokines that were increased in PBMC/K562 cultures remain to be further elucidated. It might be due to NK-induced activation of the remaining populations of mononuclear cells (monocytes and remaining subsets of lymphocytes) that were present in PBMC cultures. Alternatively, the increased levels of IL-1β, IL-1Rα, IL-6 and -8, GRO, MCP-1, MCP-3, PDGF-AA, and PDGF-AA/BB in PBMC/K562 cultures might illustrate a novel pattern of soluble factors produced by activated NK cells. To delineate between these possibilities, the flow cytometry-based detection of the intracellular levels of the aforementioned cytokines and chemokines in the selected populations of lymphocytes is desirable. Alternatively, culturing K562 cells with purified NK cells might help determine the NK-based cytokine/chemokines profile.

Importantly, the vast majority of the soluble factors that were increased in PBMC/K562 cultures after taking AgePro exhibit potent immunomodulatory properties and therefore might potentiate the cytotoxic activities of NK- and CD8-lymphocytes (e.g., TNF-α, IFN-γ, IL-1β), facilitate antigen presentation, and enhance the homing and chemotaxis of the diverse subpopulations of immune cells (e.g., GRO, MCP-1, MCP-3, etc.), etc. Two soluble factors exhibit opposite activities among 26 cytokines/chemokines that were elevated in PBMC/K562 supernatants. This includes IL-10 and IL-1Rα (interleukin-1 receptor antagonist). Indeed, like TGF-β, IL-10 was known as an immunosuppressive cytokine that limits an excessive NK cell activation by dampening the secretion of IL-12, -15, and -18 by antigen-presenting cells [64,65]. However, several studies also illustrated the immunomodulatory activity of IL-10. For example, in the presence of IL-12 and -18, IL-10 stimulated NK cell proliferation, cytotoxic activity, and IFN-γ production via activation of STAT3 signaling pathway [64,66,67]. Similarly, the upregulation of anti-inflammatory IL-1Rα was proposed to play a protective role in cytokine-mediated toxicities [68].

Collectively, we show herein the significant enhancement of the cytotoxic activity of NK cells against target tumor cells after taking AgePro for 1 month. This was associated with the increased release of several cytokines and chemokines (e.g., TNF-α, IL-1β, IL-6 and -8, MCP-1 and -3, and MIP-1α), involved in the regulation of a broad spectrum of mechanisms outside the cell-mediated cytotoxicity and killing of target cells. A strong correlation pattern was found between NK-based cytotoxic activity and the production of TNF-α, IL-1β, IL-6, and MIP-1α. Of note, no evidence of spontaneous (e.g., in the absence of target cells) activation of PBMCs, particularly NK cells, was detected after taking AgePro. Given that spontaneous activation of autoreactive lymphocytes is a feature associated with autoimmunity and taking into account our current data illustrating the AgePro-induced activation of NK cells detected only in the presence of the malicious and potentially harmful (e.g., cancerous) cells, we conclude that our innovative product exhibits potent immunoregulatory activity and high safety profile.

We acknowledge several limitations that should be considered when interpreting our findings. They are the following: (1) Small sample size and population diversity—our study included 17 healthy participants with a broad age range (23–89 years), representing a relatively small and heterogeneous cohort. While this diversity allows us to observe AgePro’s effects across different age groups, it also limits the statistical power and generalizability of our findings. Future studies with larger, more stratified cohorts will be necessary to confirm our results and identify potential age- or sex-specific variations in response. (2) Lack of placebo-controlled, double-blind design—although we observed significant increases in NK cell cytotoxicity and cytokine production after AgePro consumption, the absence of this type of study design limits our ability to rule out potential placebo effects or natural immune fluctuations. Future randomized controlled trials (RCTs) are required to validate these findings. (3) Limited scope of immune assessment—we focused on NK cell cytotoxicity, cytokine secretion, and immune activation markers. However, the immune system is highly complex, and our study did not evaluate potential effects on adaptive immunity (T and B cells), regulatory pathways, or immune-metabolic interactions.

## 4. Materials and Methods

This study involved 17 individuals (9 women and 8 men). The participants ranged in age from 23 to 89, with the largest group comprising individuals aged between 23 and 35 (53%). The mean age was 45.8 ± 18.0 years. The respondents voluntarily agreed to participate in the study and freely provided written, informed consent before participation.

### 4.1. Blood Sample Preparation

Peripheral blood was collected in heparinized tubes from 17 healthy volunteers (8 men and 9 women aged 23 to 89). Peripheral blood was used for determining the hematological parameters of the participants, including the following: red blood cells (RBCs), white blood cells (WBCs), hemoglobin (HGB), hematocrit (HCT), mean corpuscular volume (MCV), mean corpuscular HGB (MCH), mean corpuscular HGB concentration (MCHC), RBCl distribution width coefficient of variation (RDW-CV), RBC distribution width standard deviation (RDW-SD), platelets (PLTs), mean platelet volume (MPV), platelet distribution width by volume (PDW), platelet large cell ratio (P-LCR), platelet crit (PCT), immature granulocytes (IG), neutrophils (Neu), lymphocytes (Lym), monocytes (Mon), eosinophils (Eos), basophils (Bas), nucleated RBCs (NRBCs), and also erythrocyte sedimentation rate (ESR) (Westergren method (as shown in Supplementary Table S5). The hematological parameters were measured on a Cube 30 touch (DIESSE Diagnostica Senese S.p.A. Società Benefit, Italy) and Sysmex XN 1000 (Sysmex, Kobe, Japan).

PBMCs were used for the NK cytotoxicity, CD107a degranulation, and activation assays. Human PBMCs were purified from healthy adult participants using density-gradient centrifugation with Ficoll–Hypaque (d = 1.077) (Paneco, Moscow, Russia), washed twice with phosphate-buffered saline (PBS), and further used for the aforementioned assays, as shown below. For negative and positive controls, PBMCs were either unstimulated or stimulated for 12 h with mitogen phytohaemagglutinin (PHA) (Paneco, Moscow, Russia) (final concentration of 5 μg/mL). PHA-induced activation of PBMCs was assessed by flow cytometry due to the increased expression of CD25 and HLA-DR molecules as the markers of lymphocyte activation.

### 4.2. Cells and Cell Culture

Human myelogenous leukemia K562 cells were obtained from the American Type Culture Collection (ATCC, Manassas, VA, USA) and cultured in Roswell Park Memorial Institute (RPMI) 1640 medium (Paneco, Moscow, Russia) supplemented with 10% heat-inactivated fetal bovine serum (Gibco, Grand Island, NY, USA), 100 U/mL penicillin, and 100 μg/mL streptomycin (Paneco, Moscow, Russia) in a humidified atmosphere of 5% CO_2_ at 37 °C (LamSystems, Myass, Russia).

### 4.3. Antibodies

The antibodies used for flow cytometry were as follows: FITC- and PE-conjugated anti-human HLA-DR mAb and PE/Cy7-conjugated anti-human CD25 mAb (Biolegend, San Diego, CA, USA), APC-conjugated anti-human CD3 mAb, FITC-conjugated anti-human CD56 mAb, and PE-conjugated anti-human CD107 mAb (BD Biosciences, Franklin Lakes, NJ, USA).

### 4.4. PBMC NK Cytotoxicity Assay

The PBMC NK cytotoxicity assay using K562 cells was performed as previously described [23]. Briefly, K562 target cells were labeled with carboxyfluorescein diacetate succinimidyl ester (CFSE; Lumiprobe Life Science Solutions, Moscow, Russia) for 10 min at 37 °C in the dark and washed twice with complete culture medium to quench the labeling reaction. CFSE is a stable, protein-binding fluorescent dye that allows discrimination of target cells from effector NK cells [24,69]. CFSE-stained K562 cells (0.5 × 10^5^) were transferred to FACs tubes and co-cultured with the effector cells (PBMC) at 6:1, 12:1, 25:1, and 50:1 effector: target (E:T) ratios for four hours at 37 °C in a humidified 5% CO_2_ incubator. Finally, mixed cells were labeled with 1 μL of 1 mg/mL propidium iodide (PI) (Sigma-Aldrich, St. Louis, MI, USA) and analyzed by FACSCanto II cytometer (Becton Dickinson Biosciences, Franklin Lakes, NJ, USA) to determine the percentage of dead (e.g., PI-positive) CFSE-positive target cells according to the manufacturer’s protocols. BD FACSDiva Software, version 7.0 was used to analyze the samples.

### 4.5. CD107a Degranulation Assay

To analyze NK cell degranulation, their CD107a expression was measured according to the protocol, as described previously [53,70]. Briefly, 1 × 10^7^ PBMCs were mixed with 1 mL of RPMI medium and cultured alone (negative control) or in the presence of IL-2 (20 ng/mL) (ProSpec-Tany TechnoGene Ltd., Ness-Ziona, Israel) (positive control) for four hours at 37 °C in a humidified 5% CO_2_ incubator. A total of 1 × 10^7^ PBMCs was also cultured in 200 μL of complete culture medium with 10 μL PE-conjugated anti-CD107a mAb (BD Biosciences, Franklin Lakes, NJ, USA) in the absence or presence of 2 × 10^5^ K562 cells. Monensin (2 μM; eBiosciences, San Diego, CA, USA) was introduced into the cell culture following a one-hour incubation, and cells were further incubated for an additional three hours. Thereafter, cells were stained with APC-conjugated anti-human CD3 mAb and FITC-conjugated anti-human CD56 mAb (BD Biosciences, Franklin Lakes, NJ, USA) for 25 min at room temperature in the dark. Cells were washed with PBS and analyzed on a FACSCanto II cytometer (BD Biosciences, Franklin Lakes, NJ, USA).

### 4.6. Multiplex Analysis of Cytokines

A 41-multiplex analysis of an array of granules (MILLIPLEX MAP Human cytokine/chemokine magnetic bead panel, Merck, Kenilworth, NJ, USA) was used to examine the levels of cytokines/chemokines in PBMC supernatants. MAGPIX^®^ reader (Bio-Rad, Hercules, CA, USA) was used with xPONENT software, version 4.2 (Luminex Corp., Austin, TX, USA). The Luminex MAGPIX^®^ instrument was previously calibrated with the MAGPIX^®^ Calibration Kit (EMD Millipore, Merck, Kenilworth, NJ, USA); performance was verified with the MAGPIX^®^ Performance Verification Kit (EMD Millipore, Merck, Kenilworth, NJ, USA). MILLIPLEX^®^ Analytes were used to count the results and optimize the performance of the MAGPIX reader.

### 4.7. Statistics

Data were statistically processed in R software, version 4.0.2 (R Foundation for Statistical Computing, Vienna, Austria; URL https://www.R-project.org/ (accessed on 26 December 2024).

The Shapiro–Wilk test was used to assess the normality in the sample distribution. Normally distributed data are presented as mean ± standard deviation, whereas data not normally distributed are presented as median (quant 25; quant 75).

Statistical significance between two dependent samples was determined by paired Student’s *t*-test (in the case of normally distributed data) or Wilcoxon signed-rank test (in the case of not normally distributed data). The r-value was used to interpret the value of the effect in the Wilcoxon signed-rank test. Interpretation of effect size r according to Cohen: r < 0.1 illustrates no or minimal effect, 0.1–0.3—small effect, 0.3–0.5—moderate effect, 0.5–1—high effect.

The parametric statistical method (Welch Two Sample *t*-test) determined statistical significance between two normally distributed independent samples. A nonparametric statistical method assessed statistical significance between two independent samples that were not normally distributed (Wilcoxon–Mann–Whitney test).

Statistical significance between more than two experimental groups was determined by one-way analysis of variance (ANOVA). Pairwise comparisons were then performed with the Benjamini-Hochberg (BH) correction.

A two-tailed Fisher’s exact test was used to examine the significance of the relationship between two variables in a contingency table (in particular, between cytokines production and NK-based cytotoxic activity). φ criterion was used to interpret the strength of the relationship. Interpretation of the φ criterion values according to the recommendations of Rea and Parker: <0.1—the strength of the relationship is insignificant, 0.1–0.2—weak, 0.2–0.4—average, 0.4–0.6—relatively strong, 0.6–0.8—strong, 0.8–1.0—very strong.

Spearman’s rank correlation was used to determine the relationship between two non-normally distributed variables. The correlation’s strength was interpreted according to r value: r value between 0 and 0.1 indicates no correlation, between 0.1 and 0.3—low correlation, between 0.3 and 0.5—moderate correlation, between 0.5 and 0.7—potent correlation, r value between 0.7 and 1 indicates robust correlation.

The differences were considered significant at *p* < 0.05.

## Figures and Tables

**Table 1:** The killing of K562 target (T) cells by PBMC effector (E) cells was calculated for different E:T ratios before (Day 1) and after taking AgePro (Day 30). % Inc—% increase the killing of K562 after taking AgePro.

Criteria		Avg Age	Day 1	Day 30	% Inc	*p*	r
E:T ratio 50:1	All	45.9 ± 18.0	41.1 ± 19.1	54 ± 19.3	31%	0.0008	
	Age <= 35	32 ± 3.6	42.7 ± 17.5	55.3 ± 19.3	30%	0.03	
	Age >= 50	61.5 ± 14.2	39.3 ± 21.8	52.5 ± 20.5	34%	0.02	
E:T ratio 25:1	All	45.9 ± 18.0	21.9 (18.7–32.7)	39.2 (31.5–50.5)	79%	0.03	0.46
	Age <= 35	32 ± 3.6	21.9 (20.2–30.1)	40.1 (30.1–50.9)	83%	0.008	0.47
	Age >= 50	61.5 ± 14.2	28.6 ± 17.4	39.0 ± 9.81	36%	0.08 ns	
E:T ratio 12:1	All	45.9 ± 18.0	13.3 (8.9–19.7)	27.3 (20.9–34.7)	105%	0.00002	0.58
	Age <= 35	32 ± 3.6	13.3 (10.9–18.5)	27.3 (23.2–33.0)	105%	0.004	0.68
	Age >= 50	61.5 ± 14.2	15.6 ± 12.6	27.3 ± 8.15	75%	0.0009	
E:T ratio 6:1	All	45.9 ± 18.0	6.2 (4.6–9.7)	18.1 (15–21.3)	192%	0.00008	0.72
	Age <= 35	32 ± 3.6	6.2 (4.6–8.7)	19.3 (15.9–24.7)	211%	0.004	0.84
	Age >= 50	61.5 ± 14.2	7.6 (2.0–14.3)	17.3 (13.6–18.9)	128%	0.04	0.45

The Shapiro–Wilk test was used to assess the normality in the sample distribution. Normally distributed data is presented as mean ± standard deviation, whereas data not normally distributed is presented as median (quant 25; quant 75). *p*-value was used to calculate the differences between normally distributed samples by the parametric statistical method (paired Student’s test); r-value was used to interpret the value of the effect between samples that were not normally distributed. This was assessed using a nonparametric statistical method (Wilcoxon signed-rank test). r < 0.1 illustrates no effect/very small effect, r = 0.1—small effect, r = 0.3 moderate effect, r = 0.5—high effect. The differences were considered significant at *p* < 0.05, ns—nonsignificant.

**Table 2:** Expression of CD107a, the degranulation marker, on PBMCs cultured alone (negative control), in the presence of IL-2 (positive control), K562 target cells, or a combination of IL-2 and K562 cells (n = 17).

CD107a Expression	PBMC	PBMC + IL-2	PBMC + K562	PBMC + K562 + IL-2
Mean	3.09	4.34	6.77	6.80
Standard deviation	0.91	1.42	2.08	2.08
% increased relative to PBMC	-	41%	120%	120%
*p*	-	0.13	0.007	0.02

Statistical significance between the experimental groups was determined by one-way analysis of variance (ANOVA). To determine the increase in CD107a-positive cells (in %) in the aforementioned experimental groups when compared to PBMCs alone (e.g., negative control), Benjamini–Hochberg (BH) correction was used. The differences were considered significant at *p* < 0.05.

**Table 3:** The levels of cytokines/chemokines (pg/mL) in supernatants of PBMCs cultured with K562 cells before (Day 1) and after taking AgePro (Day 30).

Cytokine	Day 1	Day 30	% Inc	*p*	r
IL-1β	32.8 ± 15.6	56.9 ± 27.4	74%	0.044	
IL-1Rα	77.9 ± 52.0	199 ± 93.7	156%	0.0025	
IL-4	89.4 (75.2–98.0)	118 (102–124)	32%	0.0024	0.54
IL-6	92.7 (61.4–128)	220 (74.1–313)	137%	0.027	0.4
IL-7	6.45 ± 2.23	8.61 ± 2.12	34%	0.032	
IL-8	1766 ± 1044	3645 ± 1253	106%	0.0024	
IL-10	14.4 (9.94–30.2)	34.8 (29.8–45.7)	142%	0.012	0.45
IL-12p40	13.0 ± 4.79	19.6 ± 7.19	51%	0.0296	
IL-12p70	4.99 ± 1.42	6.90 ± 1.16	38%	0.0066	
IFN-γ	11.5 ± 5.34	16.3 ± 6.41	41%	0.054 ns	
TNF-α	292 (202–509)	720 (551–784)	147%	0.012	0.52
TNF-β	3.2 (3.2–3.34)	3.86 (3.34–4.43)	21%	0.014	0.53
G-CSF	31.8 (25.6–36.8)	43.6 (38.4–51.9)	37%	0.0068	0.59
GM-CSF	9.61 ± 3.25	12.6 ± 2.09	31%	0.024	
EGF	109 ± 36.2	150 ± 43.5	38%	0.011	
FGF-2	90.4 (88.1–94.0)	106 (103–112)	17%	0.00098	0.74
PDGF-AA	355 (290–415)	576 (355–857)	62%	0.027	0.37
PDGF-AB/BB	7921 (6514–9160)	10,588 (9800–11,888)	34%	0.0049	0.55
VEGF	41.1 ± 7.2	49.6 ± 7.66	21%	0.0054	
sCD40L	335 ± 230	412 ± 400	23%	0.48 ns	
GRO	1946 ± 1079	3418 ± 1630	76%	0.0075	
MCP-1	1254 (765–1766)	3297 (2647–6300)	163%	0.0068	0.61
MIP-1α	1301 ± 862	2638 ± 891	103%	0.0041	
MCP-3	55.7 (37.0–60.9)	126 (97.8–232)	126%	0.0015	0.72
Eotaxin	26.2 (24.5–30.5)	33.3 (30.9–36.9)	27%	0.00098	0.54
MDC	118 ± 39.6	163 ± 49.6	38%	0.014	

The Shapiro–Wilk test was used to assess the normality in the sample distribution. Normally distributed data is presented as mean ± standard deviation, whereas data not normally distributed is presented as median (quant 25; quant 75). *p*-value was used to calculate the differences between normally distributed samples by the parametric statistical method (paired Student’s test); r-value was used to interpret the value of the effect between samples that were not normally distributed. This was assessed using a nonparametric statistical method (Wilcoxon signed-rank test). r < 0.1 illustrates no effect/very small effect; r = 0.1—small effect; r = 0.3—moderate effect; r = 0.5—high effect. The differences were considered significant at *p* < 0.05, ns—nonsignificant.

**Table 4:** Spearman’s rank correlation between NK-based cytotoxicity and cytokine/chemokines production.

Cytokine/Chemokine	Spearman’s Rank Correlation rho (r)	*p*
MIP-1a	0.769697	0.014 *
TNF-alpha	0.6969697	0.031 *
IL-6	0.7575758	0.016 *
IL-1beta	0.4060606	0.247 ns

Spearman’s rank correlation was used to determine the relationship between two non-normally distributed variables. The correlation’s strength was interpreted according to r value: r value between 0 and 0.1 indicates no correlation, between 0.1 and 0.3—low correlation, between 0.3 and 0.5—moderate correlation, between 0.5 and 0.7—potent correlation, r value between 0.7 and 1 indicates robust correlation. The differences were considered significant at *p* < 0.05 (*), ns—relationship was not significant.

**Table 5:** The numbers of CD56/107a-positive cells (in %) in PBMCs cultured alone (control) or in the presence of K562 target cells.

	Day 1		Day 30		Day 1/Day 30	
	PBMC	PBMC + K562	PBMC	PBMC + K562	PBMC	PBMC + K562
Mean ± SD	1.95 (1.84–2.06)	8.78 (7.96–9.45)	2.46 (1.88–3.2)	18.40 (16.3–20.7)	-	-
Fold increase	-	4.5	-	7.5	1.2	2.1
*p*	-	0.0022	-	0.0022	0.44 ns	0.031
r						0.83

The Shapiro–Wilk test was used to assess the normality in the sample distribution. Not normally distributed data is presented as median (quant 25–quant 75). *p*-value of the effect between two independent samples was assessed by a nonparametric statistical method (Wilcoxon–Mann–Whitney test). Statistical significance between two dependent samples was determined by the Wilcoxon signed-rank test. The r-value was used to interpret the value of the effect in the Wilcoxon signed-rank test. r < 0.1 illustrates no or minimal effect, 0.1–0.3—small effect, 0.3–0.5—moderate effect, 0.5–1—high effect. The differences were considered significant at *p* < 0.05, ns—nonsignificant.

## Data Availability

Data is contained within the article and Supplementary Materials.

## Supplementary Materials

The following supporting information can be downloaded at: https://www.mdpi.com/article/10.3390/ijms26072897/s1.
