# Supplementary material for: Enhancement of NK Cell Cytotoxic Activity and Immunoregulatory Effects of a Natural Product Supplement Across a Wide Age Span: A 30-Day In Vivo Human Study
Source: Int J Mol Sci. 2025 Mar 22;26(7):2897. doi: 10.3390/ijms26072897 (PMC11988361; doi:10.3390/ijms26072897)
Supplement: Supplementary file 1 [file ijms-26-02897-s001.zip › ijms-3488030-supplementary.pdf]

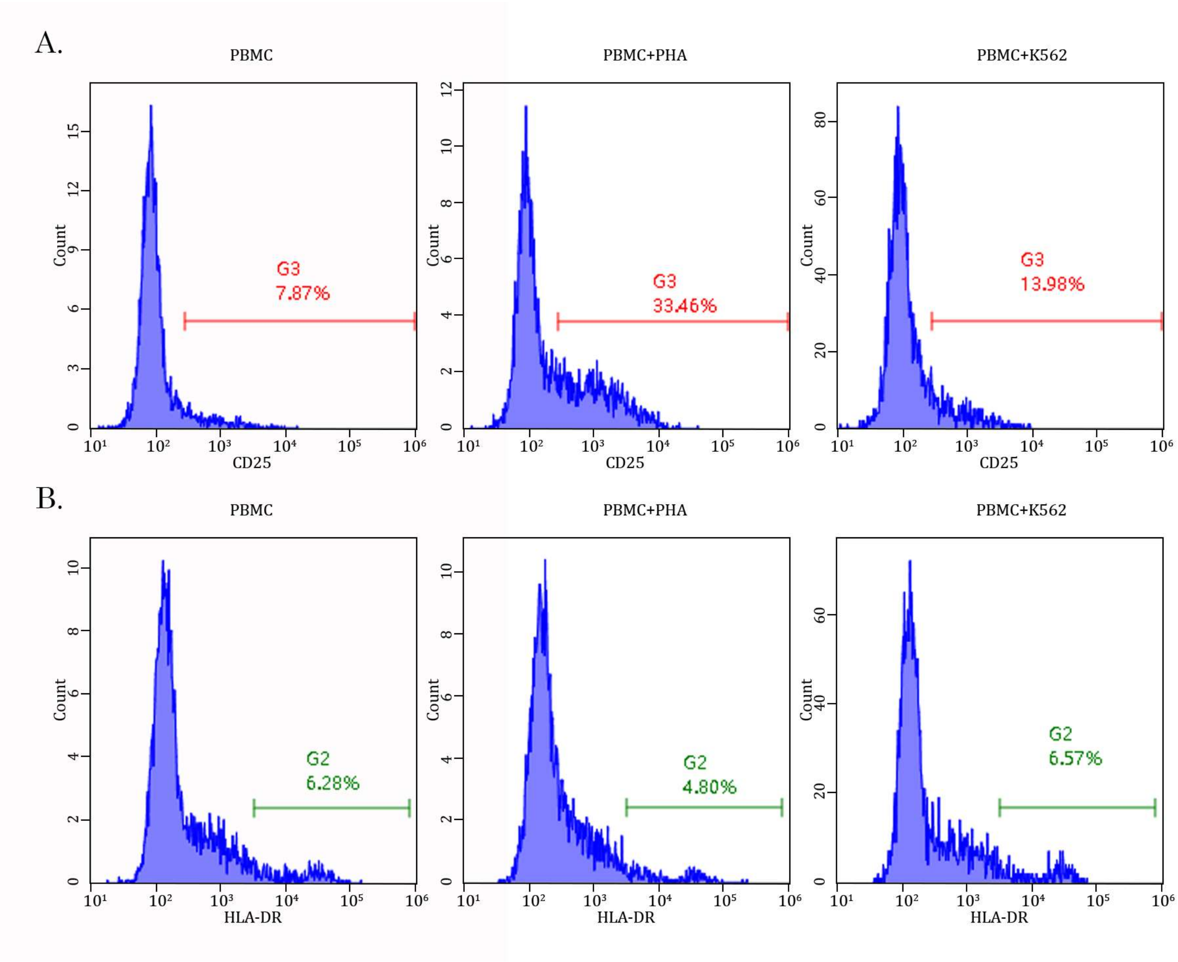

Supplementary Figure S1. Representative histograms illustrating the expression of CD25 (**A**) and HLA-DR (**B**) activation markers in PMBC cultured alone (**left**), in the presence of K562 target cells (**right**) or stimulated with PHA (5 μg/ml) (**middle**) for 12 h.

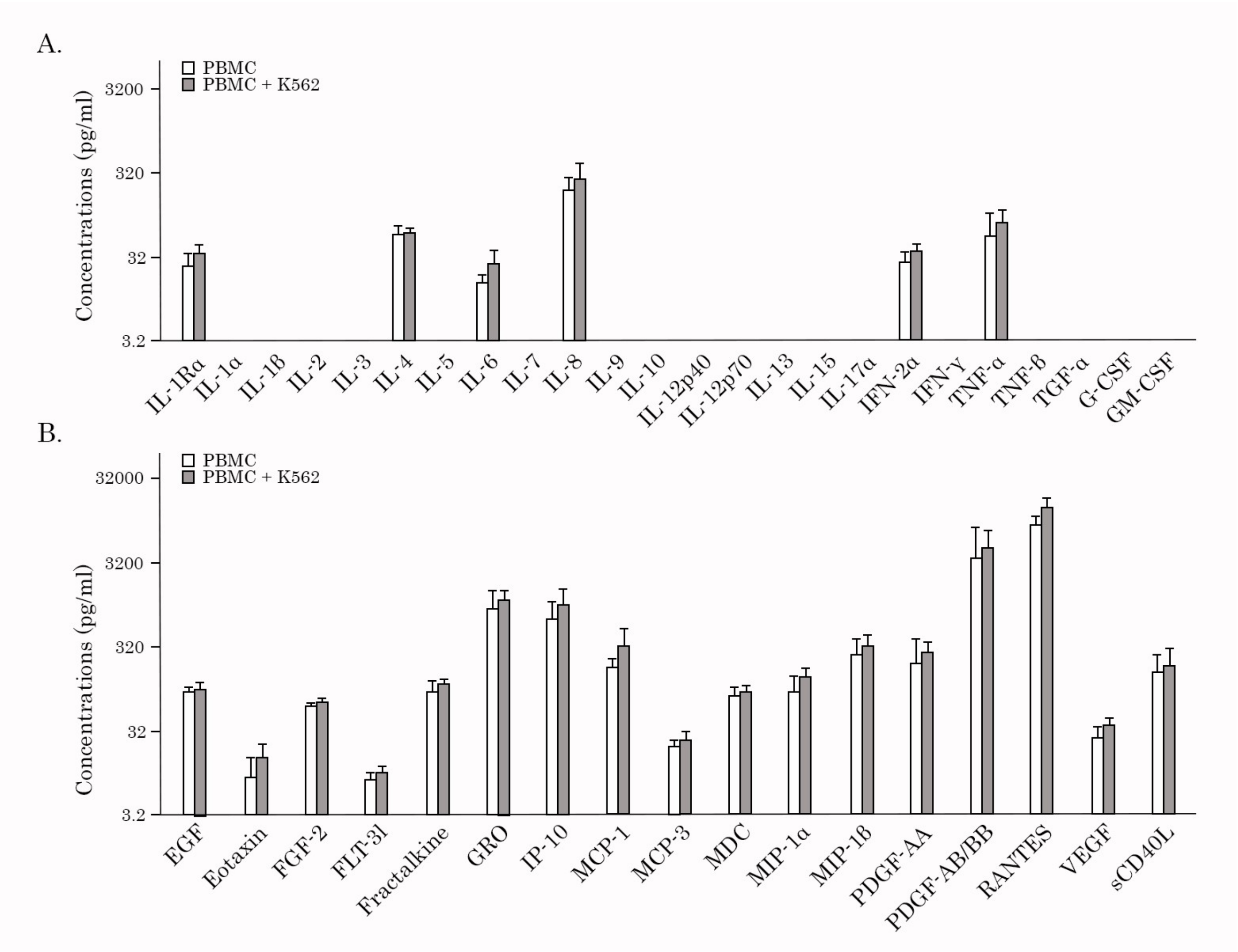

Supplementary Figure S2. The concentration of cytokines and chemokines (pg/ml) in the supernatants of PBMC cultured alone before (gray columns) and after (white columns) taking AgePro. (**A**) IL-1Rα, -1α, -1β, -2, -3, -4, -5, -6, -7, -8, -9, -10, -12p40, -12p70, -13, -15, -17α, INF-2α, INF-γ, TNF-α, TNF-β, TGF-α, G-CSF, and GM-CSF; (**B**) EGF, eotaxin, FGF-2, FLT-3l, fractalkine, GRO, IP-10, MCP-1, MCP-3, MDC, MIP-1α, MIP-1β, PDGF-AA, PDGF-AA/AB, RANTES, VEGF, and sCD40L.

Supplementary Table S1. The levels of cytokines/chemokines (pg/ml) in supernatants of PBMC cultured alone and in the presence of K562 target cells.

Cytokine	PBMC	PBMC+K562	Fold increase	p
**IL-1Rα**	26.5(16.5-27.2)	91.2(69.2-113)	3.44	0.0097
**IL-1β**	<3.2	32.8±15.6	10.25	
**IL-4**	61.9±12.8	85.6 ±28.1	1.38	0.031
**IL-6**	16.4(16.3-17.8)	92.7(61.4-128)	5.65	0.0019
**IL-7**	<3.2	6.45±2.23	2.02	
**IL-8**	162(154-169)	1449(1073-2565)	8.94	0.0019
**IL-10**	<3.2	20.9±16.6	6.53	
**IL-12p40**	<3.2	13±4.79	4.06	
**IL-12p70**	<3.2	4.99±1.42	1.56	
**INF-2α**	31.3(29.4-33)	45.9(44.8-51.6)	1.47	0.0071
**IFN-γ**	<3.2	11.6±5.34	3.63	
**TNF-α**	43.2(41.5-62.2)	292(202-509)	6.76	0.0019
**TNF-β**	<3.2	3.3±0.16	1.03	
**G-CSF**	<3.2	28.8±13.3	9.00	
**GM-CSF**	<3.2	9.61±3.25	3.00	
**EGF**	87.5(82.7-99.8)	104(95.4-131)	1.19	0.19 ns
**Eotaxin**	9.45(3.2-13.4)	26.2(24.5-30.5)	2.77	0.007
**FGF-2**	62.0(62.0-66.0)	90.4(88.1-94)	1.46	0.0095
**FLT-3l**	8.4(7.74-9.03)	19.1(18.2-21.1)	2.27	0.0052
**Fractalkine**	107(107-107)	154(125-162)	1.44	0.014
**GRO**	725(711- 1246)	1848(1349-2170)	2.55	0.02
**IP-10**	600(499-746)	1807(1082-3638)	3.01	0.1 ns
**MCP-1**	197(145-210)	1254(765-1766)	6.37	0.0098
**MCP-3**	21.6(18.3-23.5)	55.7(37.0-60.9)	2.58	0.0098
**MDC**	84.4±19.5	118±39.6	1.40	0.033
**MIP-1α**	106(62.8-133)	1190(665-1635)	11.23	0.0019
**MIP-1β**	258(245-322)	5787(3020-10000)	22.43	0.0065
**PDGF-AA**	205±198	362±183	1.77	0.17 ns
**PDGF-AB/BB**	3564±4772	7143±3341	2.00	0.18 ns
**RANTES**	9204(6856-10000)	12004(10527-13630)	1.30	0.058 ns
**VEGF**	28.0±7.02	41.1±7.25	1.47	0.009
**sCD40L**	183(145-201)	357(118-499)	1.95	0.19 ns

The Shapiro–Wilk test was used to assess the normality in the sample distribution. Normally distributed data is presented as mean ± standard deviation, whereas data not normally distributed is presented as median (quant 25; quant 75). *p*-value was used to calculate the differences between normally distributed samples by the parametric statistical method (Welch Two Sample t-test); the *p*-value of the effect between samples that were not normally distributed was assessed by a nonparametric statistical method (Wilcoxon–Mann–Whitney test). The differences were considered significant at $p < 0.05$, ns - nonsignificant.

Supplementary Table S2. The numbers of CD25- HLA-DR-positive cells (in %) in PBMC cultured alone (control) or in the presence of K562 target cells or stimulated with PHA (5 μg/ml) for 12 h.

	**CD25-positive cells**			**HLA-DR-positive cells**		
	**PBMC**	**PBMC+PHA**	**PBMC+K562**	**PBMC**	**PBMC+PHA**	**PBMC+K562**
Mean	11.20	31.00	18.30	6.73	4.86	7.04
Standard deviation	4.28	9.13	3.93	1.73	1.31	1.25
% increased relative to PBMC	-	177%	63%	-	-68%	5%
P	-	0.030	0.049	-	0.28 ns	0.79 ns

Statistical significance between the experimental groups was determined by one-way analysis of variance (ANOVA). To determine the increase of CD25 or HLA-DR-positive cells (in %) in the aforementioned experimental groups when compared to PBMC alone (i.e., negative control), Benjamini–Hochberg (BH) correction was used. The differences were considered significant at $p < 0.05$, ns – nonsignificant.

Supplementary Table S3. The relationship between cytokine/chemokines secretion (< 50% / > 50% increase after taking AgePro) and NK-based cytotoxic activity (group 1 with RR < 20% / group 2 with RR > 20%).

	**Group 1 (n=4)**	**Group 2 (n=8)**	**p**	**φ**
**IL-8**				
< 50% Inc	3 (25%)	1 (12.5%)	0.067 ns	
> 50% Inc	1 (75%)	7 (87.5%)		
**MCP-1**				
< 50% Inc	1 (75%)	2 (25%)	1.0 ns	
> 50% Inc	3 (25%)	6 (75%)		
**MIP-1a**				
< 50% Inc	4 (100%)	1 (12.5%)	0.0101	0.837
> 50% Inc	0 (0%)	7 (87.5%)		
**TNF-a**				
< 50% Inc	3 (25%)	0 (0%)	0.018	0.816
> 50% Inc	1 (75%)	8 (100%)		
**IL-6**				
< 50% Inc	4 (100%)	1 (12.5%)	0.0101	0.837
> 50% Inc	0 (0%)	7 (87.5%)		
**MCP-3**				
< 50% Inc	0 (0%)	2 (25%)	0.52 ns	
> 50% Inc	4 (100%)	6 (75%)		
**IL-1Ra**				
< 50% Inc	3 (25%)	2 (25%)	0.22 ns	
> 50% Inc	1 (75%)	6 (75%)		
**IL-10**				
< 50% Inc	2 (50%)	2 (25%)	0.13 ns	
> 50% Inc	2 (50%)	6 (75%)		
**IL-1b**				
< 50% Inc	4 (100%)	2 (25%)	0.0303	0.707
> 50% Inc	0 (0%)	6 (75%)		
**IL-12p40**				
< 50% Inc	2 (50%)	3 (37.5%)	1.0 ns	
> 50% Inc	2 (50%)	5 (62.5%)		
**PDGF-AA**				
< 50% Inc	1 (75%)	4 (50%)	0.58 ns	
> 50% Inc	3 (25%)	4 (50%)		
**GRO**				
< 50% Inc	1 (75%)	3 (37.5%)	1.0 ns	
> 50% Inc	3 (25%)	5 (62.5%)		

Interpretation of the φ criterion values according to the recommendations of Rea & Parker: <0.1 – the strength of the relationship is nonsignificant, 0.1-0.2 – minimal, 0.2-0.4 – moderate, 0.4-0.6 – relatively strong, 0.6-0.8 – potent, 0.8-1.0 – very strong. The differences were considered significant at $p < 0.05$, ns - nonsignificant.

Supplementary Table S4. The levels of cytokines/chemokines (pg/ml) in supernatants of PBMC before (day 1) and after (day 30) taking AgePro.

Cytokine	Day 1	Day 30	% Inc	p
IL-1Rα	25±9.97	35.1±8.95	1.40	0.102 ns
IL-4	61.9±12.8	64.1 ±6.1	1.04	0.705 ns
IL-6	16.4(16.3-17.8)	21.6(20.2-31.3)	1.31	0.063 ns
IL-8	162(154-169)	150(192-228)	0.93	0.063 ns
INF-2α	31.3(29.4-33)	41.6(33.5-44.4)	1.33	0.19 ns
TNF-α	57.7±48.5	83.9±32.6	1.45	0.33 ns
EGF	91.7±14	101±15.4	1.10	0.32 ns
Eotaxin	9.45(3.2-13.4)	17.4(17.1-19.5)	1.84	0.31 ns
FGF-2	62.0(62.0-66.0)	76.6(71.5-76.6)	1.23	0.063 ns
FLT-3l	8.6±1.42	9.6±2.86	1.11	0.44 ns
Fractalkine	107(107-107)	129(111-132)	1.20	0.31 ns
GRO	898±558	1144±355	1.27	0.18 ns
IP-10	685±394	1006±538	1.47	0.051 ns
MCP-1	197(145-210)	241(223-518)	1.22	0.44 ns
MCP-3	21.3±4.21	25.2±7.08	1.18	0.054 ns
MDC	80.3(80.3-93.2)	94.7(82-108)	1.18	0.063 ns
MIP-1α	106(62.8-133)	135(131-153)	1.27	0.13 ns
MIP-1β	258(245-322)	322(259-336)	1.25	0.502 ns
PDGF-AA	205±198	281±96.9	1.37	0.23 ns
PDGF-AB/BB	3564±4772	4780±3158	1.34	0.21 ns
RANTES	8786±2336	10990±6845	1.25	0.44 ns
VEGF	28.0±7.02	38.5±6.24	1.38	0.092 ns
sCD40L	183(145-201)	210(195-239)	1.15	0.13 ns

The Shapiro–Wilk test was used to assess the normality in the sample distribution. Normally distributed data is presented as mean ± standard deviation, whereas data not normally distributed is presented as median (quant 25; quant 75). p-value was used to calculate the differences between normally distributed samples by the parametric statistical method (paired Student's test); the *p*-value of the effect between samples that were not normally distributed was assessed by a nonparametric statistical method (Wilcoxon signed-rank test). The differences were considered significant at $p < 0.05$, ns - nonsignificant.

Supplementary Table S5. Hematological parameters of the participants before (day 1) and after (day 30) taking AgePro.

Test	Reference values	Unit of measurement	Day 1	Day 30	p
RBC	3.70 - 4.70 (f)	x10^{12}/l	4.52±0.18	4.45±0.25	0.38 ns
	4.00 - 5.10 (m)		5.1(4.76-5.1)	5.1(4.70-5.1)	1 ns
WBC	4.00 - 9.00	x10^{9}/l	6.88±1.2	6.28±0.79	0.056 ns
HGB	120 – 150 (f)	g/l	132.75±7.98	131±7.65	0.23 ns
	130 – 170 (m)		151.75±9.45	151.29±10.63	0.93 ns
HCT	35.0 - 47.0 (f)	%	39.04±2.48	38.41±2.05	0.22 ns
	39.0 - 50.0 (m)		43.21±2.34	43.47±2.76	0.68 ns
MCV	80 - 100	fl	85.67±3.72	86.29±4.18	0.50 ns
MCH	27.0 - 32.0	pg	29.48±1.25	29.65±1.2	0.25 ns
MCHC	315 – 356 (f)	g/l	340.25±2.76	341.63±7.03	0.68 ns
	320 – 370 (m)		353(348-355)	354(338-357)	0.67 ns
RDW-CV	11.20 - 15.60	%	13.05±0.87	12.97±0.8	0.45 ns
RDW-SD	35.2 - 51.6	fl	40.61±3.77	40.44±3.24	0.75 ns
PLT	180 - 400	x10^{9}/l	299.2±51.26	289.13±37.5	0.34 ns
MPV	7.4 - 10.4	fl	9.8(9.55-10.2)	9.8(9.4-10.3)	0.96 ns
PDW	11.2 - 15.6	fl	11.5(11.2-11.9)	11.2(11.2-12.6)	0.76 ns
P-LCR	13.0 - 43.0	%	24.32±5.43	24.17±5.45	0.81 ns
PCT	0.15 - 0.40	%	0.3±0.05	0.29±0.04	0.28 ns
IG	0.00 - 0.04	x10^{9}/l	0.02(0.02-0.035)	0.02(0.01-0.02)	0.066 ns
Neu	1.90 - 6.50	x10^{9}/l	3.54±0.83	3.12±0.61	0.059 ns
Lym	0.80 - 3.30	x10^{9}/l	2.38±0.57	2.28±0.48	0.51 ns
Mon	0.08 - 0.81	x10^{9}/l	0.57±0.1	0.57±0.12	0.96 ns
Eos	0.02 - 0.45	x10^{9}/l	0.24(0.18-0.35)	0.18(0.165-0.34)	0.088 ns
Bas	0.00 - 0.12	x10^{9}/l	0.06±0.03	0.05±0.03	0.24 ns
NRBC	0.00 - 0.03	x10^{9}/l	0(0-0)	0(0-0)	1 ns
ESR	0 – 20 (f)	mm/h	9.13±4.16	10.38±2.83	0.30 ns
	0 – 15 (m)		4.16±1.46	7.43±3.78	0.052 ns

The Shapiro–Wilk test was used to assess the normality in the sample distribution. Normally distributed data is presented as mean ± standard deviation, whereas data not normally distributed is presented as median (quant 25; quant 75). p-value was used to calculate the differences between normally distributed samples by the parametric statistical method (paired Student's test); the *p*-value of the effect between samples that were not normally distributed was assessed by a nonparametric statistical method (Wilcoxon signed-rank test). The differences were considered significant at $p < 0.05$, ns - nonsignificant.
